# Repeated subsamples during DNA extraction reveal increased diversity estimates in DNA metabarcoding of Malaise traps

**DOI:** 10.1002/ece3.9502

**Published:** 2022-11-27

**Authors:** Vera M. A. Zizka, Matthias F. Geiger, Thomas Hörren, Ameli Kirse, Niklas W. Noll, Livia Schäffler, Alice M. Scherges, Martin Sorg

**Affiliations:** ^1^ Leibniz Institute for the Analysis of Biodiversity Change (LIB), Zoological Research Museum Alexander Koenig (ZFMK) Centre for Biodiversity Monitoring and Conservation Science Bonn Germany; ^2^ Entomological Society Krefeld (EVK) Krefeld Germany

**Keywords:** arthropod metabarcoding, biodiversity, DNA extraction, monitoring

## Abstract

With increased application of DNA metabarcoding in biodiversity assessment, various laboratory protocols have been optimized, and their further evaluation is subject of current research. Homogenization of bulk samples and subsequent DNA extraction from a subsample of destructed tissue is a common first stage of the metabarcoding process. This can either be conducted using sample material soaked in a storage fixative, e.g., ethanol (here referred to as “wet” treatment) or from dried individuals (“dry”). However, it remains uncertain if perfect mixing and equal distribution of DNA within the tube is ensured during homogenization and to what extent incomplete mixing and resulting variations in tissue composition affect diversity assessments if only a fraction of the destructed sample is processed in the downstream metabarcoding workflow. Here we investigated the efficiency of homogenization under wet and dry conditions and tested how variations in destructed tissue composition might affect diversity assessments of complex arthropod samples. We considered five time intervals of Malaise trap bulk samples and process nine different subsamples of homogenized tissue (20 mg each) in both treatments. Results indicate a more consistent diversity assessment from dried material, but at the cost of a higher processing time. Both approaches detected comparable OTU diversity and revealed similar taxa compositions in a single tissue extraction. With an increased number of tissue subsamples during DNA extraction, OTU diversity increased for both approaches, especially for highly diverse samples obtained during the summer. Here, particularly the detection of small and low‐biomass taxa increased. The processing of multiple subsamples in the metabarcoding protocol can therefore be a helpful procedure to enhance diversity estimates and counteract taxonomic bias in biodiversity assessments. However, the process induces higher costs and time effort and the application in large‐scale biodiversity assessment, e.g., in monitoring schemes needs to be considered on project‐specific prospects.

## Graphical abstract

Different tissue homogenisation protocols in DNA metabarcoding of Malaise trap samples are compared and the effect of higher tissue amounts during DNA extraction is investigated. Wet homogenisation of tissue is recommended due to a low risk of cross‐contamination, while the processing of more tissue during extraction increases detected insect diversity by up to 30%, especially for species rich samples.

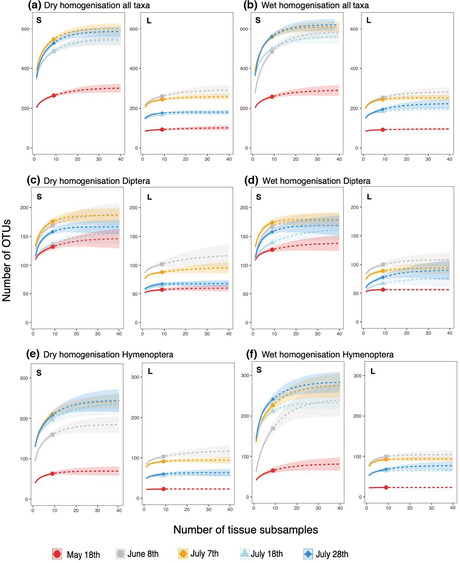

## 1 INTRODUCTION

For highly diverse groups such as terrestrial insects, where morphological identification is difficult, slow, and expensive, DNA metabarcoding provides an efficient alternative (Bush et al., 2019; Evans et al., 2016; van der Heyde et al., 2020; Yu et al., 2012). In recent years, the method has been the focus of several studies, which evaluated and discussed promising sampling strategies (Gleason et al., 2020; Marquina et al., 2019; Pereira‐da‐Conceicoa et al., 2021; Steinke et al., 2020), laboratory procedures (Elbrecht et al., 2019; Majaneva et al., 2018; Piñol et al., 2019; Zizka, Elbrecht, et al., 2019; Zizka, Leese, et al., 2019), bioinformatic analyses (Frøslev et al., 2017; Porter & Hajibabaei, 2020; Turon et al., 2020), and ways of integration into existing biodiversity monitoring matrices (Buchner et al., 2019; Cordier et al., 2018; Mächler et al., 2021; Pawlowski et al., 2018). This has resulted in a variety of different DNA metabarcoding protocols, with standardization still lacking. However, streamlining workflows is essential to ensure comparability of data from different studies and the use of metabarcoding for applied biodiversity monitoring (Bush et al., 2019; McGee et al., 2019; Pawlowski et al., 2018).

DNA can be extracted from enclosed communities, including the sample's fixative ethanol (Marquina et al., 2019; Zenker et al., 2020) or propylene glycol (Martoni et al., 2021), from added lysis buffer (Batovska et al., 2021; Giebner et al., 2020; Kirse et al., 2021) or from homogenized tissue of specimens (Hardulak et al., 2020; Mata et al., 2021; Zizka et al., 2020). While the latter approach is currently considered the most efficient in biodiversity assessment (Hardulak et al., 2020; Marquina et al., 2019; Persaud et al., 2021; Zenker et al., 2020; Zizka, Elbrecht, et al., 2019), it prevents subsequent morphological identifications as source material is destroyed (Nielsen et al., 2019). Homogenization and tissue‐based DNA extraction can be conducted from wet samples in ethanol (Beentjes et al., 2019; Pereira‐da‐Conceicoa et al., 2021; Porter et al., 2019) or from dried tissue after ethanol evaporation (Elbrecht et al., 2019; Hardulak et al., 2020; Hausmann et al., 2020; Steinke et al., 2020). Since drying and homogenizing result in a fine powder, it requires more careful handing than wet material due to the increased chance of cross‐contamination. Most DNA extraction protocols are limited to low amounts of starting tissue per reaction, and only a subsample of complete material is usually processed, ranging between 1 and 100 mg (Hausmann et al., 2020; Marquina et al., 2019; Mata et al., 2021; Sire et al., 2022). Higher tissue volume during DNA extraction requires multiple reactions or more voluminous DNA extraction kits resulting in an increased effort and cost. However, DNA extraction from the subsampled tissue makes the assumption of perfect homogenization and equal distribution within storage tubes, and it remains unknown to what extent variation in tissue composition affects the assessment of species contained within bulk samples.

The effect of different extraction protocols has been examined in a number of studies, but the majority of which are either based on aquatic samples or do not include an evaluation of the pre‐extraction steps (Majaneva et al., 2018; Mata et al., 2021; Pereira‐da‐Conceicoa et al., 2021). The material from these studies constitutes a lower diversity and biomass than typical Malaise trap samples, which means they cannot be directly compared as the latter samples require additional adjustments. Buchner and Leese (2020) investigated the overlap of species detection between subsamples of homogenized tissue obtained from Malaise traps; here, the authors focused on wet homogenization. In the current study, we provide a detailed examination of the effect dry homogenization and an examination into the different taxonomic groups in the assessed communities. We use five time‐interval Malaise trap samples collected in a protected area in Germany and investigate the effect of homogenization strategy and tissue subsampling on biodiversity assessments. This study provides new insights in efficiency of extraction protocols for Malaise trap sampling and introduces a way of increasing diversity detection for tissue‐based DNA metabarcoding approaches.

## 2 MATERIALS AND METHODS

### 2.1 Sampling

Samples were collected in the Nature reserve “Latumer Bruch” near Krefeld in Western Germany. All samples originate from one Malaise trap (51.326701 N, 6.632973 E). Detailed information about samples taken between May and July is given in Table 1.

**TABLE 1:** Malaise trap samples analyzed: shown is the sampling interval and wet biomass (g)

ID	Sampling interval	Wet biomass (g)
T 1	12.05–18.05.19	14.1
T 2	29.05–08.06.19	65.9
T 3	28.06–07.07.19	71.7
T 4	07.07–18.07.19	40
T 5	18.07–28.07.19	70.1

Malaise trap sampling was conducted in a standardized manner based on the bicolored model by Henry Townes (Matthews & Matthews, 1983; Townes, 1972). A detailed description of the Malaise trap setup with photographic illustrations can be found in the supplementary material of Hallmann et al. (2017). Insects were captured in 96% denatured ethanol (1% MEK). After bottle collection, ethanol was replaced with new 96% undenatured ethanol and stored at −20°C for further processing.

### 2.2 Laboratory work

**FIGURE 1:**
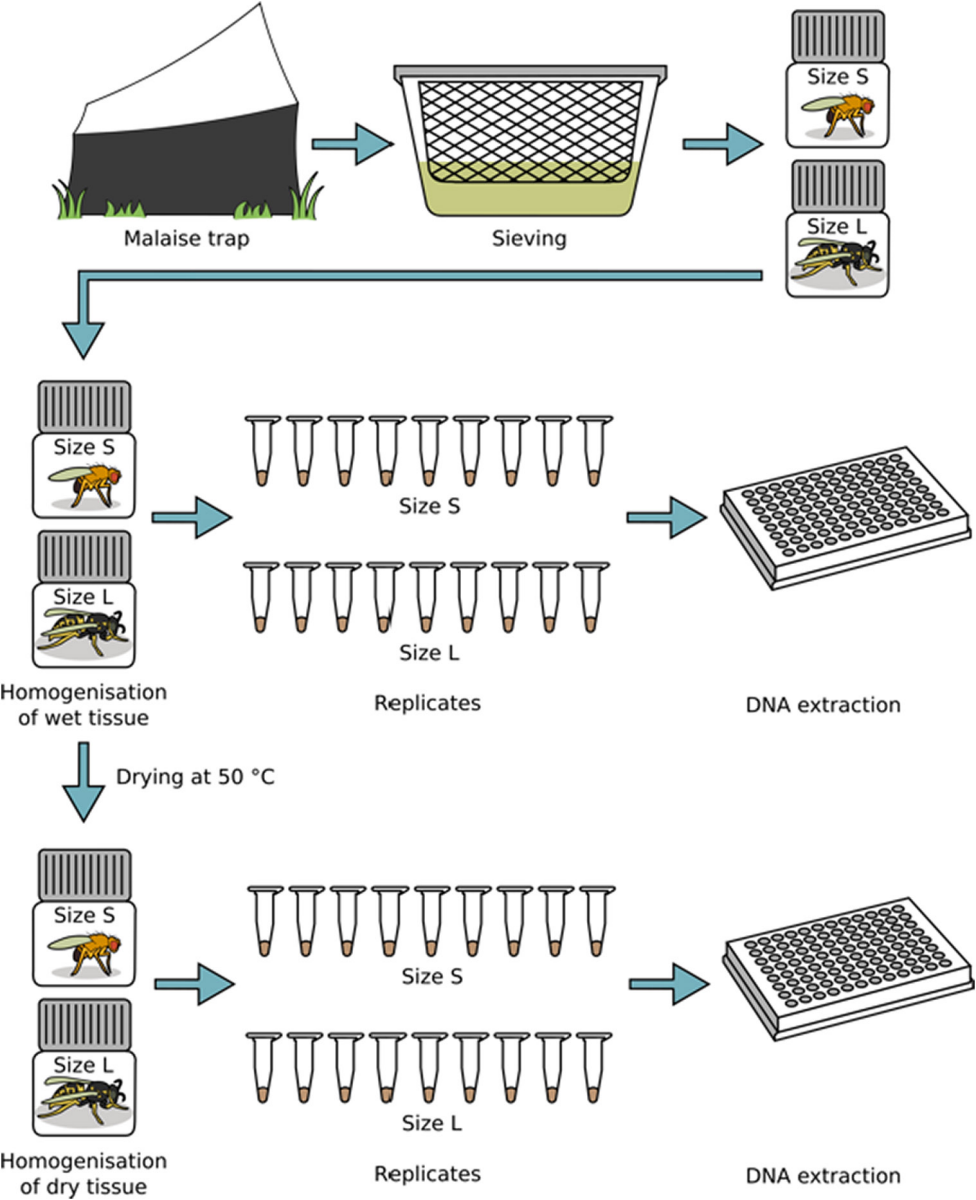
Experimental setup. Malaise trap samples were sieved in ethanol and separated into size fraction **L**arge (>4 mm) and **S**mall (≤4 mm). Wet tissue was homogenized, and nine subsamples per size fractions with ~20 mg each were transferred to 1.5 μl Eppendorf tubes for separate DNA extraction. Homogenized tissue was dried and again homogenized in dry conditions. Again, nine subsamples per size fraction with ~20 mg each were transferred to 1.5 μl Eppendorf tubes. Subsequent DNA workflow was conducted as described in Material and Methods.

Supernatant ethanol was removed, and each sample was separated into two size classes by sieving wet specimens through a 4 mm × 4 mm mesh with a wire diameter of 0.5 mm (untreated stainless steel). In the following, the size fractions will be referred to as either S (small, ≤4 mm) or L (large, >4 mm). Depending on sample volume, individuals of both size classes were transferred to either 30 ml tubes (Nalgene, wide‐mouth bottle, polypropylene) or 50 ml Falcon tubes, and approximately 20 g of grinding balls (5 mm diameter, stainless steel, Retsch GmbH) was added to each sample. Both size fractions per sample were processed separately throughout the whole metabarcoding protocol and kept separate in bioinformatic analysis (Figure 1). Homogenization of wet samples was conducted with a mixer mill MM 400 (Retsch GmbH) at a frequency of 30 s^−1^ for 5 min. Nine subsamples per sample and size class were transferred to 1.5 ml Eppendorf tubes in wet condition, centrifuged for 1 min at 10,000 rpm (Heraeus Fresco 21 Centrifuge; Thermo Scientific), and dried overnight in a shaking incubator (ILS6, VWR) at 50°C (90 samples in total, five samples × two treatments × nine subsamples). Subsamples were weighed (20 mg ± 6 mg) on a fine scale balance (Entris, Sartorius). Together with six negative controls (200 μl ATL lysis buffer [Qiagen, Hilden, Germany], no sample added, kept through the whole laboratory protocol and sequencing), DNA was extracted with the DNeasy 96 Blood and Tissue Kit (Quiagen) following manufacturer instructions. Remaining sample tissue (in 30 ml tubes, not transferred to 1.5 Eppendorf tubes) was centrifuged for 1 min at 4700 rpm (MegaStar 1.6, VWR Collection), and the supernatant was discarded afterward. Remaining tissue was left to dry in a shaking incubator at 50°C for up to 3 days until complete ethanol evaporation. Dried tissue of samples containing <30 ml was again homogenized at a frequency of 30 s^−1^ for 5 min with the Retsch mixer mill (MM400). Samples consisting of more than 30 ml source material were homogenized for 3 min with a Turax mixer mill (Tube Mill 100 Control) at 25,000 rpm because dried material was clustered to a hard unit and could not be destructed with the former mill. Nine subsamples per sample were transferred to 1.5 Eppendorf tubes and weighed (23 mg ± 5 mg). To ensure processing of identical samples during this experiment, already homogenized samples under wet treatment were dried and homogenized again under dry conditions. This included an additional homogenization step for those samples. The former approach will be referred to as *wet homogenization*, while the latter approach (wet with additional dry homogenization) will be referred to as *dry homogenization*. Together with six negative controls (200 μl ATL lysis buffer [Qiagen], no sample added, kept through the whole laboratory protocol and sequencing), DNA was extracted with the DNeasy 96 Blood and Tissue Kit (Quiagen) following manufacturer instructions for both plates. Extraction success and DNA quality were checked on a 1% agarose gel.

A two‐step PCR protocol was applied using standard Illumina Nextera primers for dual indexing of samples. The first PCR (PCR 1) was performed with the PCR Multiplex Plus Kit (Qiagen) using 12.5 μl master mix, 1 μl of DNA template, 0.2 μM of the fwhF2 forward (Vamos et al., 2017), and Fol_degen_rev reverse (Yu et al., 2012) primers, respectively. The primer pair targets a 313 bp long stretch of the COI DNA barcode region and ensures a sufficient overlap of fragments during paired‐end merging after 2 × 250 bp sequencing. Additionally, this primer pair was positively evaluated in a comprehensive comparison of Malaise trap samples (Elbrecht et al., 2019). The PCR mix was filled with 10.5 μl ddH_2_O to a 25 μl reaction volume. The following PCR program was applied: initial denaturation at 95°C for 5 min; 25 cycles of: 30 s at 95°C, 30 s at 50°C, and 50 s at 72°C; final extension of 5 min at 72°C. First step PCR (PCR 1) product was used for the second PCR (PCR 2), also conducted with the PCR Multiplex Plus Kit (Qiagen). Dual indexing of sequences was applied to guarantee the assignment of sequences to the sample of origin in bioinformatic analysis. A set of 12 forward and eight reverse primers (per sequencing run) with unique identifiers (MID) integrated was used to tag sequences of samples with a unique index combination. Indexes were integrated between sequencing primer binding site and Illumina sequencing adapter following the Nextera XT Index Kit v2 for library preparation. The following tags were used: Miseq run 1 (samples dry homogenization), forward MID: TCGCCTTA, CATGCCTA, AGCGTAGC, CAGCCTCG, TAGCGAGT, TACTACGC, AGGCTCCG, GCAGCGTA, CTGCGCAT, GTCTTAGG, TAGCTGCA, GACGTCGA; reverse MID: CTCTCTAT, TCGACTAG, TTCTAGCT, CCTAGAGT, CTATTAAG, AAGGCTAT, GAGCCTTA, TTATGCGA. Miseq run 2 (samples wet homogenization), forward MID: CTAGTACG, TTCTGCCT, GCTCAGGA, AGGAGTCC, TGCCTCTT, TCCTCTAC, TCATGAGC, CCTGAGAT, GTAGCTCC, GAGCGCTA, CGCTCAGT, ACTGATCG; reverse MID: GCGTAAGA, TCTCTCCG, CGTCTAAT, CTAAGCCT, AAGGAGTA, ACTGCATA, GTAAGGAG, TATCCTCT. Reaction included 1 μl DNA template from PCR 1, 0.2 μM of each tagging primer (Nextera, Illumina), and 12.5 μl master mix filled up with 10.5 μl H_2_O. Primers included a nucleotide overhang as a binding site for primers in PCR 2, which was run with the same program as PCR 1 but with only 15 cycles. PCR success was evaluated on a 1% agarose gel before PCR products were normalized using a SequalPrep Normalization plate (Thermo Fisher Scientific) following the manufacturer's instructions with an end concentration of 25 ng per sample (100 μl). For each sample, 10 μl was pooled together, and left‐sided size selection with magnetic beads was applied twice on the sample pool to remove primer residuals (ration 0.76×, SPRIselect Beckman Coulter). Library concentration was measured with a Quantus fluorometer (Promega) and on a FragmentAnalyzer (Agilent Technologies), and the pool was sent for sequencing on two Miseq runs (2 × 300 bp) to Macrogen Europe B.V.

### 2.3 Data analysis

The quality of sequences delivered by Macrogen was determined through the program Fastqc (Andrews et al., 2012). Subsequent data processing was conducted using standard settings for all samples as implemented in JAMP v0.67 (https://github.com/VascoElbrecht/JAMP). Paired‐end reads were merged with vsearch v2.15.0 (Rognes et al., 2016). Cutadapt v3.4 (Martin, 2011) was used to remove primers and to discard sequences of unexpected length so that only reads with a length of 303–323 bp were used for further analyses. All reads with an expected error >0.5 were excluded from further analysis. Sequences were dereplicated, singletons were removed, and sequences with ≥97% similarity were clustered into Operational Taxonomic Units (OTUs) using uparse. Chimera filtering was conducted using the uchime3_denovo option in vsearch. OTUs with a minimal read abundance of 0.003% per sample were retained for further analysis, and the program LULU was used for further qualitative filtering (Frøslev et al., 2017). Reads in negative controls were subtracted from according OTUs in samples (processed on the same extraction plate and sequenced on the same sequencing run). Taxonomic assignment of OTUs was conducted by comparison with a custom Arthropoda reference database. The database was created using *taxalogue* (https://github.com/nwnoll/taxaloguecommit:62ce71819af40a6e605e9142f0ccd69318477596) with sequences and taxonomies obtained from BOLD Systems (BOLD) (https://www.boldsystems.org), GenBank (NCBI) (https://www.ncbi.nlm.nih.gov/genbank/) and the German Barcode of Life (GBOL) (https://bolgermany.de/gbol1/ergebnisse/results). Taxon names were normalized according to the NCBI Taxonomy (https://www.ncbi.nlm.nih.gov/taxonomy). Only OTUs that had at least 85% similarity with a sequence in a reference database were retained for further analysis (using vsearch version 2.14.1 with ‐usearch_global command and the following parameters: ‐id 0.85, ‐dbmask none, ‐qmask none, ‐maxhits 1000, blast6out and ‐maxaccepts 0). Custom scripts (available at: https://github.com/nwnoll/homogenisation_replicates) were used to extract the best hits and assign each OTU a taxonomic name. Statistical analyses were conducted in R version 4.0.2 (R Core Team, 2017). Non‐metric multidimensional scaling (NMDS) was performed based on Jaccard (presence/absence) and Bray–Curtis (abundance) dissimilarity matrices using the R package vegan (v2.5‐6) (Oksanen et al., 2019). To assess dissimilarity within and between treatments, a Permutational Multivariate Analysis of Variance (PERMANOVA) was applied, using vegan (v2.5‐6). For analysis of differences in OTU numbers between homogenization methods, size fractions, and subsamples, a one‐way Analysis of Variance (ANOVA) was performed, followed by a Kruskal–Wallis test as integrated with the package stats (v4.0.2) (R Core Team, 2017). For species accumulation curves and associated calculations of extrapolated values, the package iNEXT (v.2.0.2) (Hsieh et al., 2016) was used. Figures were constructed with the package ggplot2 (v3.3.6) (Wickham, 2016).

## 3 RESULTS

On the two Miseq runs, 13.3 and 14.7 million reads in forward and reverse direction respectively were assigned to the specified index combinations. Raw data are uploaded at NCBI Short Read Archive (accession number: PRJNA883590). Extraction negative controls for samples processed with dry homogenization included on average 86 ± 82 reads after OTU clustering, while negative controls for samples processed with wet homogenization included on average 520 ± 561 reads. Reads were extracted from OTUs in regarding samples, processed on the same extraction plate and sequencing run. After quality filtering, on average, 114,394 (±20,365) reads were kept per sample.

For both homogenization approaches combined and all subsamples, in total 1528 OTUs were clustered with the following order‐level assignment: Coleoptera 158 (10.3%), Diptera 372 (24.3%), Hemiptera 134 (8.8%), Hymenoptera 689 (45.1%), Lepidoptera 52 (3.4%), other orders 98 (6.4%), no assignment to order level 25 (1.6%). Of those, 1088 OTUs were assigned to genus or species level. Detailed information about OTU assignments and read distribution on order level is summarized in Table 2 and Table S1. While the highest number of OTUs was assigned on average to Diptera (Large size fraction >4 mm: 41.6%, Small size fraction ≤4 mm: 37.5%) and Hymenoptera (L: 38%, S: 32.3%), the main proportion of reads was related to Diptera (L: 60.8%, S: 77%) and <10% to Hymenoptera (Table 2). This was most pronounced for size fraction S, where on average only 4.7% of the reads were assigned to this highly diverse order. A fixed threshold of 97% similarity was used for OTU clustering, and several OTUs show the same species‐level assignments (Table S1). Further analyses were based on total OTU numbers, and no merging of molecular units with identical species‐level assignment was conducted. We choose this diversity level to circumvent the merging of OTUs only assigned to higher taxonomic levels (as genus or family) since we have no information if those belong to the same species. Since the comprehensiveness of reference databases is strongly biased toward specific groups and shows huge gaps for others (Geiger et al., 2016), the lumping of OTUs based on assigned taxonomy could lead to a group‐specific underestimation of diversity.

**TABLE 2:** Average proportion of OTUs and reads assigned per time interval and through all samples to the orders Coleoptera, Diptera, Hemiptera, Hymenoptera, Lepidoptera, other arthropod orders and OTUs not assigned to order level (see Table S1) for both homogenization approaches and all subsamples combined. Both size classes were processed and analyzed separately, here defined in column “Size”, L = size fraction large including specimens >4 mm, and S = size fraction small including specimens ≤4 mm.

Order	Size	OTU number (%)	Read number (%)	Total OTU number
Coleoptera	L	5.7 ± 1.9	6 ± 4.9	158 (10.3%)
	S	11.1 ± 1	5.5 ± 2.8	
**Diptera**	**L**	**41.6 ± 9.8**	**60.8 ± 13.3**	**372 (24.4%)**
	**S**	**37.5 ± 8.8**	**77 ± 11.9**	
Hemiptera	L	3 ± 0.8	0.4 ± 0.3	134 (8.8%)
	S	11.2 ± 2.3	10.1 ± 10.7	
**Hymenoptera**	**L**	**38 ± 5.2**	**18.7 ± 7.9**	**689 (45.1%)**
	**S**	**32.3 ± 7.5**	**4.7 ± 2.5**	
Lepidoptera	L	7.6 ± 3.2	13.8 ± 5.4	52 (3.4%)
	S	2.9 ± 1.3	1.8 ± 1	
Other arthropods	L	2.8 ± 0.8	0.3 ± 0.2	98 (6.4%)
	S	3.6 ± 0.6	0.2 ± 0.1	
No assignment	L	1.2 ± 0.4	0.1 ± 0.2	25 (1.64%)
	S	1.3 ± 0.3	0.7 ± 0.5	

**FIGURE 2:**
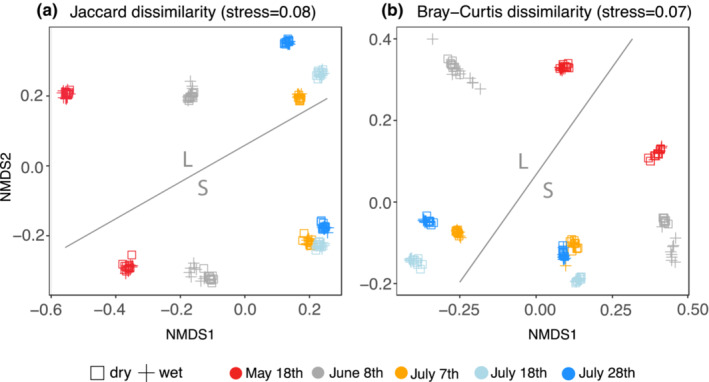
Non‐metric multidimensional scaling based on (a) Jaccard (presence/absence data) and (b) Bray–Curtis (abundance data) dissimilarity matrices. Samples include the nine subsamples per collection date (color coding), size fraction (S and L, marked in figure), and treatment during homogenization (shape coding).

While different collection dates and the different size classes per sample showed distinct community compositions (Figure 2, *p* < .002), the treatment during homogenization did not affect sample ordination in NMDS analysis (*p* = .997, Figure 2). However, the average Jaccard dissimilarity between subsamples homogenized in dry condition (0.179 ± 0.06) was lower (*p* < .001) than dissimilarities between subsamples homogenized with wet treatment (0.207 ± 0.081) and when subsamples of one emptying date and size fraction were compared among homogenization approaches (0.206 ± 0.089, *p* < .001). Bray–Curtis dissimilarity was on average lower within dry (0.11 ± 0.07) than within wet (0.11 ± 0.09) homogenized subsamples and when subsamples between the two homogenization approaches were compared (0.13 ± 0.08, *p* < .01).

**FIGURE 3:**
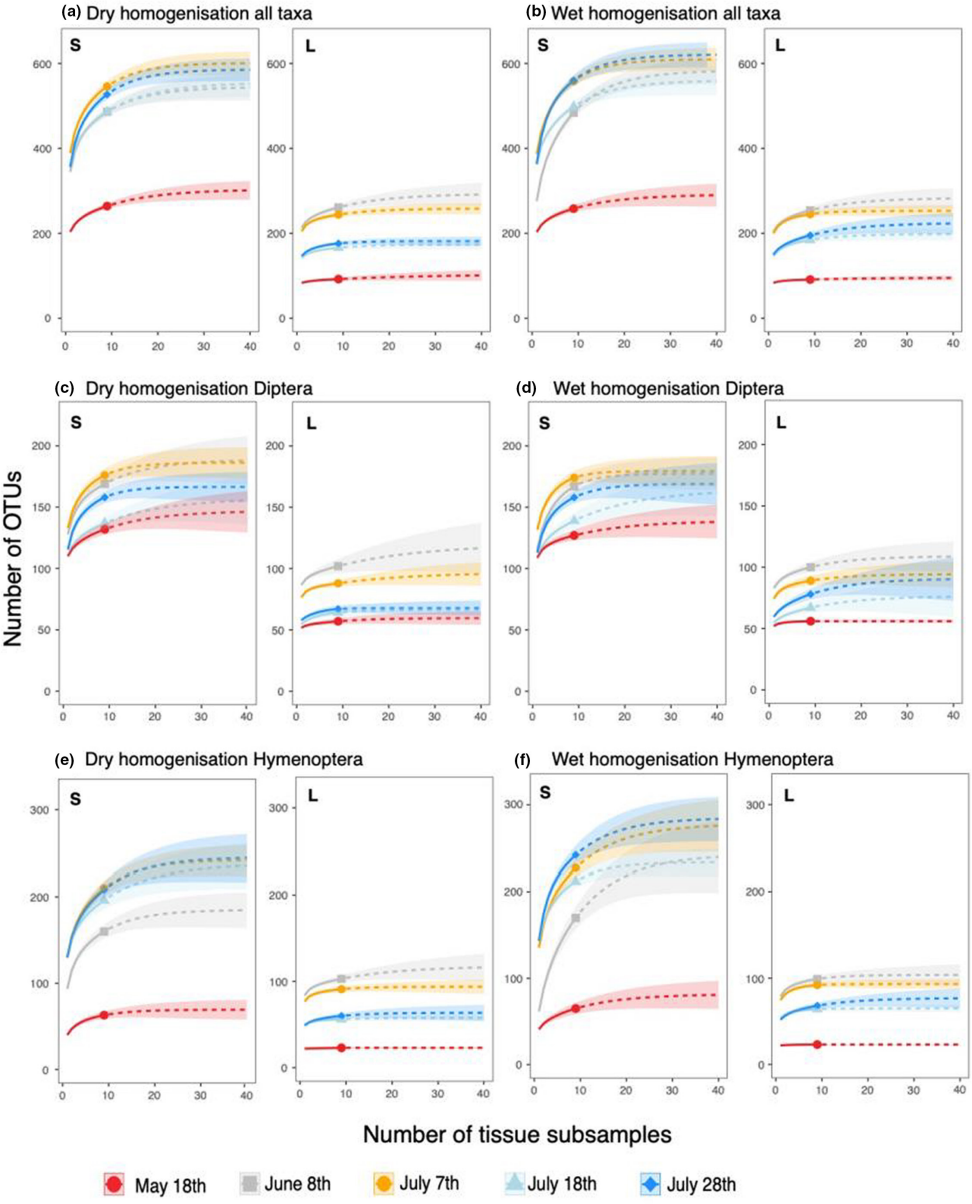
Species accumulation curves. Increased number of OTUs with increased number of subsamples (~20 mg tissue per subsample) processed in extraction is illustrated as well as extrapolation up to 40 subsamples. All taxa homogenized in (a) dry and (b) wet conditions; Diptera recovered in samples homogenized in (c) dry and (d) wet conditions; Hymenoptera detected in samples homogenized in (e) dry and (f) wet conditions. Size fraction is marked in the upper left corner of each subfigure (S = small, L = large).

For samples processed under wet treatment, on average, 60.7% ± 8.5 of extrapolated total diversity could be detected in size fraction S and 75.8% ± 7.9 in size fraction L when only one subsample was processed in extraction (~20 mg of tissue, Figure 3). With nine extraction subsamples (20 mg/subsample) 88.4% ± 3.2 and 92.4% ± 4.2 of extrapolated total richness was detected in fraction S and L respectively, while 95% of calculated species richness was assessed with 12 ± 5.4 and 17 ± 3.4 subsamples. For samples homogenized by dry treatment, a single extraction (~20 mg) of size class S revealed 64% ± 2.3 of calculated species richness. In comparison, 79% ± 3.8 could be detected in 20 mg of size class L (Figure 3). On average, 88.9% ± 1.6 of total diversity was assessed with the nine applied subsamples for size fraction S and 93.1% ± 3.8 for size fraction L. In contrast, 95% of total diversity would be calculated with 16 ± 2.1 (S, 320 mg) and 13 ± 7.1 (L, 260 mg) subsamples (Figure 3).

Detailed analysis of Hymenoptera in wet homogenized tissue revealed on average 47.2% ± 13.3 of extrapolated total species richness in size fraction S and 80.4% ± 10.4 in size fraction L when a single subsample was processed (Figure 3). Increased to nine subsamples, 81.4% ± 7.6 (S) and 96.2% ± 4.7 (L) of calculated total diversity were detected. Extrapolations revealed that 95% of total calculated diversity was achieved by processing 22 ± 5.2 (S, ~440 mg) and 7.4 ± 6 (L, ~140 mg) replicates. For dry homogenization, one extraction subsample revealed 54% ± 2.7 (S) and 82.4% ± 9.6 (L) of total species richness, while 86.2% ± 3.1 and 95.1% ± 4.8 could be assessed with nine extraction subsamples. Calculations revealed the detection of 95% of total hymenopteran species richness if 18 ± 4 (360 mg) and 9.8 ± 8 (200 mg) replicates were processed (Figure 3).

For wet homogenization, detailed analysis of Diptera representatives revealed 70.6% ± 5.6 of calculated diversity in size fraction S and 77% ± 10.3 in size fraction L when only a single extraction subsample was processed. Detected richness increased to 92.1% ± 4.7 and 91% ± 5.6 when nine subsamples were processed (95% were reached with 14.2 ± 7.9 and 5 ± 1.9 subsamples). Additional dry homogenization processing of one tissue subsample revealed 70.6% ± 2.7 of extrapolated diversity for size fraction S and 81.1 ± 5.9 for size fraction L. With nine extraction subsamples, taxa detection increased to 91.2% ± 3.4 (S) and 93.3% ± 5.5 (L). Calculations revealed detection of 95% from total Diptera if 15.6 ± 5.4 (~320 mg) and 14.2 ± 10.2 (~200 mg) replicates were processed. For detailed information about observed and calculated species richness, see Figure 3.

**FIGURE 4:**
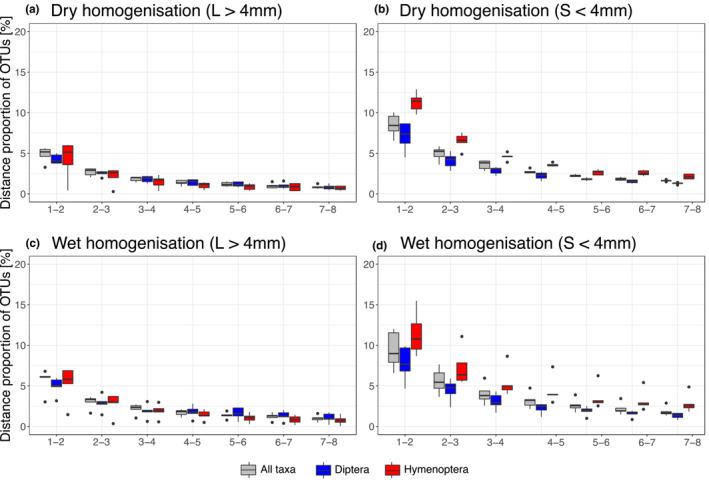
Proportion of molecular units (OTUs) detected with additional DNA extraction subsamples. (a) for dry homogenization and samples of size fraction L (>4 mm), (b) for dry homogenization and size fraction S (≤ 4 mm), (c) for wet homogenization and samples of size fraction L (> 4 mm), and (d) for wet homogenization and size fraction S (<4 mm). The *x*‐axis describes differences between processed subsamples (e.g. 1–2 difference in relative number of OTUs detected with one subsample (~20 mg) compared with two subsamples (~40 mg)).

Based on the accumulation of OTUs processing nine different subsamples (Figure 3), we calculated the proportion of additional OTUs with an increased number of subsamples (Figure 4). For both treatments, the highest increase was observed when two instead of one subsample of size fraction S (≤4 mm) were processed. The increase was the highest for OTUs assigned to Hymenoptera (average distance wet: 11.4% ± 2.7, dry: 11.3% ± 1.2). With an increased number of subsamples, the distance in OTU number between samples decreased for all treatments and size fractions.

## 4 DISCUSSION

Homogenization of bulk samples and subsequent DNA extraction from destructed tissue is widely applied when insect biodiversity is assessed using DNA metabarcoding (Beermann et al., 2021; Hardulak et al., 2020; Mata et al., 2021). This destructive approach is known to be highly efficient for bulk sample analysis at a lesser cost compared with nondestructive protocols (Marquina et al., 2019; Persaud et al., 2021; Sire et al., 2022; Zenker et al., 2020). However, the application of DNA extraction from ethanol fixative or lysis buffer has the advantage of ensuring sample integrity, and the evaluation of various protocols is ongoing. Several studies demonstrate nondestructive approaches as promising alternatives for insect diversity assessment (Carew et al., 2018; Iwaszkiewicz‐Eggebrecht et al., 2022; Nielsen et al., 2019; Svenningsen et al., 2021). A similar study based on DNA extraction from lysis buffer also demonstrated an increase in number of species detected by increasing sample volume (of buffer) during extraction (Kirse et al., 2022). However, a comparison between destructive and nondestructive approaches is out of scope of the present study. Here, we set out to test different homogenization protocols and how subsampling of homogenized tissue affects diversity estimates of highly diverse Malaise trap samples.

### 4.1 Comparison of different homogenization approaches

The average dissimilarity between reactions homogenized with dry treatment was lower than dissimilarity between subsamples processed with wet treatment for presence/absence analysis, which indicates a higher homogeneity of tissue samples processed under dry conditions. To ensure comparability and processing of identical sampling material (as stated in the Material and Methods section), the dry homogenization approach was based on material already destructed under wet conditions. This additional step of 3 min homogenization might have increased the efficiency of tissue destruction and mixing of samples and, therefore, the similarity of subsamples. In addition, on average, a lower tissue weight per subsample was processed after wet homogenization (difference between dry and wet subsamples on average 3 mg). However, Jaccard's dissimilarity indices of subsamples that were homogenized wet were on average 0.21 ± 0.08, mainly due to high inconsistencies between subsamples from June 8th with an average dissimilarity of 0.35 ± 0.02 compared with dissimilarities between subsamples of the other collection dates (0.18 ± 0.06; Figure 2). The subsample inconsistency of this collection date (8th of June) is difficult to explain. These subsamples show medium diversity estimates and more diverse samples depict a higher similarity between subsamples (July). Since we did not investigate morphological features of detected taxa, taxonomic composition could be a factor for insufficient homogenization, impacting only the wet treatment. It also needs to be considered that for the present approach, DNA quantity after extraction was only measured through the band strength on an agarose gel (band visible for all samples), and that no adjustment of DNA quantity was conducted before PCR. Since subsamples were processed from identical samples, no significant differences in extraction success due to sample composition were expected. For comparing samples constituting highly different communities, the adjustment of DNA quantity might be an option; however, the different biomass of samples also needs to be considered.

The homogenization of dried samples includes drying for at least 48 h at temperatures around 50°C to guarantee the complete evaporation of ethanol from the sample, which increases processing time of the metabarcoding protocol. While the handling of dried powder appears more sensitive to cross‐contamination than wet material processing, we could not support this with the analysis of negative controls (dry: 86 ± 82, wet: 520 ± 561). Here it needs to be considered that contamination risk was only assessed from extraction onward. For the processing of dried samples, even the drying step in a closed heating chamber could induce a contamination risk if a large number of samples is processed in parallel with insufficient spacing between samples. In comparison, the homogenization of wet samples soaked in ethanol circumvents this drying step. It is, therefore, more time‐efficient and suitable for large‐scale approaches as implemented in several studies on aquatic samples (Hajibabaei et al., 2012; Majaneva et al., 2018; Pereira‐da‐Conceicoa et al., 2021) and has been introduced as a scalable and cost‐efficient protocol elsewhere (Buchner et al., 2021). The minor differences we observed between the two applied methods and the abovementioned experimental setup allow us to recommend homogenization of wet material for tissue‐based DNA metabarcoding of Malaise trap samples as it reduces handling time and hence scalability of the protocol. Results indicate that an additional homogenization step of dried material, e.g., through bead grinding, should be integrated after the material has been subsampled to increase the fineness of material (Buchner et al., 2021).

### 4.2 Extraction subsamples during homogenization

A high but incomplete proportion of the total insect diversity was assessed when processing a single tissue subsample in extraction, which opens up an additional perspective on the basis material for homogenization (wet or dry) with variable effects on samples of different size fraction and time interval. All samples detected at least 60% of extrapolated arthropod diversity with a single extraction from 20 mg of tissue. However, analysis revealed strong differences between size fractions, time intervals, and also taxonomic groups. The processing of additional subsamples only moderately increased diversity estimates for the large size fraction and samples of both size fractions from samples collected in May. While those samples contain comparatively low biodiversity, a strong increase was detected for the remaining samples of the small size fraction collected in summer. Results indicate that especially for highly diverse samples, a higher number of subsamples during extraction, as already recommended in Elbrecht et al. (2019), or a larger tissue volume per extraction can lead to higher biodiversity values. In Geiger et al. (2016), the authors investigated individual DNA barcodes of a Malaise trap collection (>30,000 specimens, single‐specimen DNA barcoding), and almost 3000 BINs were detected within a May‐September period at one location. While this study implies a higher sampling effort (May‐September vs. May‐July) from a different habitat, it indicates that diversity estimates from a subsample depict a fraction of total Malaise trap diversity. However, additional material for extraction comes with higher expenses and project‐specific estimations need to evaluate if higher time and cost effort in the lab protocols are appropriate to obtain higher diversity estimates. While specific approaches aim to achieve complete diversity assessments or target specific taxonomic groups, the detection of 60% of total diversity is sufficient for other studies focusing on the detection of abundant taxa or analyzing the “bigger picture” of insect distribution patterns and insect decline.

The most pronounced increase in OTUs with additional extraction subsamples was observed for the order Hymenoptera. Representatives of Diptera and Hymenoptera are the main targets in many Malaise trapping studies (Ssymank et al., 2018). However, as also indicated in previous studies, Diptera are present in much higher individual numbers, constituting a higher proportion of biomass in Malaise trap catches, while diversity of both groups is considered to be similar (Geiger et al., 2016). An underrepresentation of specific insect families, especially constituting taxa of low biomass, including small as well as rare insects, has also been reported in previous metabarcoding studies (Elbrecht et al., 2019, 2021; Krehenwinkel et al., 2017; Yu et al., 2012). This includes, for example, highly diverse parasitoid Hymenoptera, which are involved in important ecosystem functions but include many tiny species contributing only a minor fraction of tissue and consequently small amounts of DNA to complex bulk sample mixtures. While insufficient primer binding efficiency is discussed as the main reason for this phenomenon, our results indicate that reduced sample volume compared with the original sample can counteract the detection of taxa constituting low biomass to the bulk sample. This has also been shown by Shirazi et al. (2021), who compared the community composition between PCR replicates, detecting only 21–29% of total species in one out of 24 reactions. While an increased diversity of especially rare taxa can be achieved by PCR replication, results indicate that complete alpha diversity of a sample can hardly be detected through eDNA metabarcoding (Shirazi et al., 2021).

In the present study, each of the nine subsamples with approximately 20 mg of homogenized tissue was processed during extraction. It needs to be further investigated how the processing of 180 mg in one reaction (e.g., through a kit tolerating higher sample volumes) affects taxa detection. Here, technical details could influence the extraction of DNA molecules from the tissue, potentially reducing the concentration of rare molecules in final reactions or affecting the downstream laboratory protocol, e.g., through high DNA quantities in PCR reactions. Additionally, higher sequencing depth could increase taxa recovery and overlap between extraction subsamples (Braukmann et al., 2019; Shirazi et al., 2021). Higher sequencing depth per sample can be reached by lower number of samples per sequencing run or more powerful sequencing platforms. This protocol adjustment also comes with a higher cost per sample, but is easier to adapt than processing multiple extraction replicates per sample. We tested a sequencing depth of on average 114,394 ± 20,365 reads per sample (after quality filtering), and detailed analysis to understand the linkage between higher sequencing depth and replication strategy is beyond the scope of the present study. Again, it is also unclear how adjustments would affect taxa detection if extraction of higher tissue volume (e.g., 180 mg) in one reaction was conducted. Further investigation could reveal an increase in sequencing depth as the most effective way to optimize taxon recovery under financial constraints, especially if one reaction of a high tissue volume is applied. Also, modifications in bioinformatic filtering can affect the detection of especially small and rare taxa, constituting low DNA amounts into the sample mixture. Quality filtering of raw sequence data as well as read assignments in OTU tables is essential to exclude PCR and sequencing errors as well as pseudogenes from the datasets and account for false‐positive assignments (Andújar et al., 2020; Piper et al., 2019; Turon et al., 2020). However, quality control often implies abundance‐based filtering, potentially excluding low‐read assignments of taxa present in the sample. Evaluating the processing of multiple subsamples in combination with data filtering adjustments would give further insights on how to optimize metabarcoding workflows for high‐resolution insect biodiversity assessment.

While the results presented here indicate an increase in insect diversity through processing a higher number of subsamples, especially for low‐biomass taxa, it needs to be considered that results are based on different time intervals of a single Malaise trap. Spreading from the middle of May to the end of July, those samples cover different magnitudes of diversity, which is also accounted for by separate processing of the different size fractions. However, a sample basis of a single trap is insufficient to formulate final recommendations for standardized DNA extraction and metabarcoding of Malaise traps. More work needs to be conducted across a variety of habitats to test the effect of different insect communities on species recovery.

## 5 CONCLUSION

We recommend homogenization of wet material in tissue‐based DNA metabarcoding of Malaise trap samples due to similar levels of taxon recovery compared with dry source material and with the advantage of lower time effort and higher scalability. In dry and wet homogenization, additional DNA extraction replicates result in a higher number of detected taxa, particularly those of low biomass and present in highly diverse samples. However, a decision on the more complete diversity detection associated with higher resources depends on the focused scientific goals of individual studies.

## AUTHOR CONTRIBUTIONS

**Vera M. A. Zizka:** Conceptualization (equal); formal analysis (lead); methodology (lead); validation (equal); visualization (equal); writing – original draft (equal); writing – review and editing (equal). **Matthias F. Geiger:** Validation (supporting); writing – review and editing (equal). **Thomas Hörren:** Conceptualization (equal); methodology (equal); validation (equal); writing – review and editing (equal). **Ameli Kirse:** Validation (supporting); visualization (equal); writing – review and editing (equal). **Niklas W. Noll:** Conceptualization (equal); formal analysis (supporting); validation (supporting); writing – review and editing (equal). **Livia Schäffler:** Conceptualization (equal); funding acquisition (equal); project administration (equal); writing – review and editing (equal). **Alice M. Scherges:** Methodology (equal); writing – review and editing (equal). **Martin Sorg:** Conceptualization (equal); funding acquisition (equal); methodology (equal); validation (equal); writing – review and editing (equal).

## CONFLICT OF INTEREST

The authors declare no competing interests.

## Supporting information


Table S1

## Data Availability

The raw sequences used in this study are available at NCBI SRA, accession number PRJNA883590.
